# Supplementary material for: Rgg144/SHP144-controlled streptolancidin D mediates intra-species competition in Streptococcus pneumoniae with cumulative effect from other bacteriocins and fratricide
Source: mBio. 2026 Aug 7;17(9):e01596-26. doi: 10.1128/mbio.01596-26 (PMC13556254; doi:10.1128/mbio.01596-26)
Supplement: Supplementary file 1 [file mbio.01596-26-s0001.pdf]

Figure S1

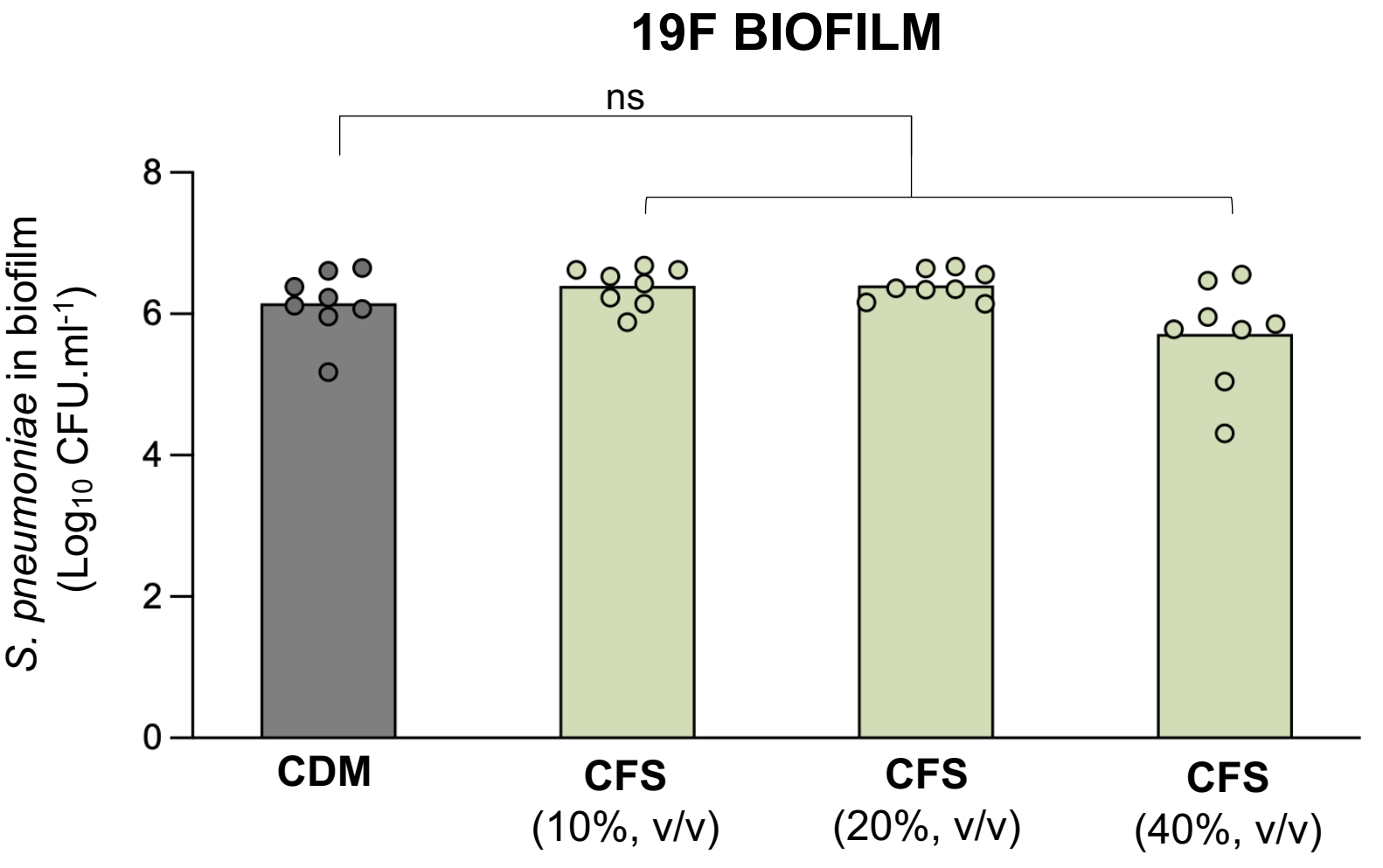

Figure S1. The cell-free supernatant of strain 19F is not inhibitory towards 19F biofilms, suggesting self-immunity against secreted molecules. Biofilms were grown in CDM for 24h and increasing concentrations of 19F cell-free supernatant (CFS) obtained from biofilm cultures were added at time of inoculation (10%, 20%, 40%, v/v). CDM, no addition of supernatant. Events.mL^{-1} were calculated based on cell counts by flow cytometry of resuspended 19F biofilms. Plot represents the results of four independent experiments, each with intra-experiment duplicates. Black bars indicate geometric means. ns., non-significant, Mann-Whitney U test with the Benjamini-Hochberg correction for false discovery rate.

Figure S2

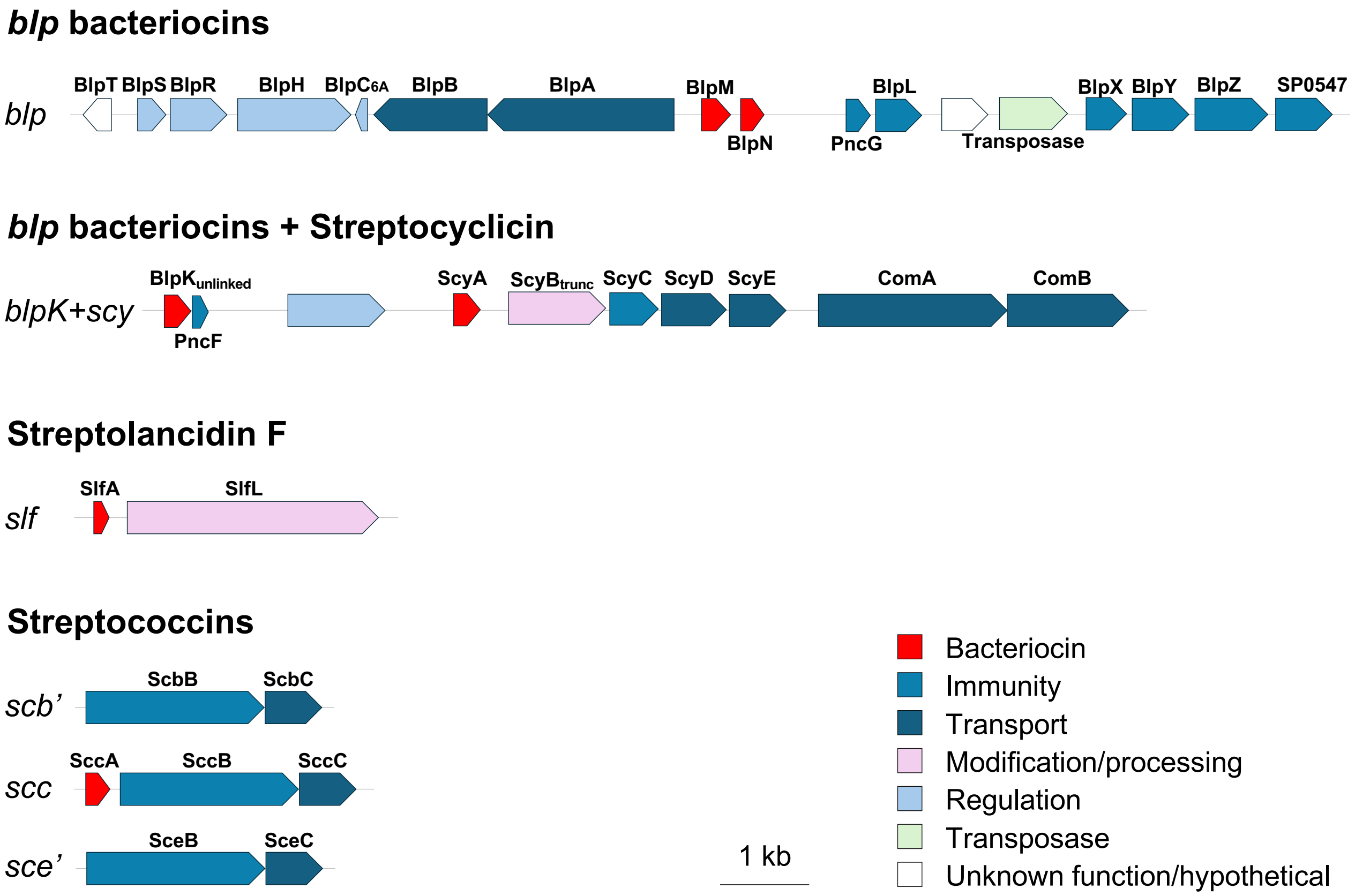

Figure S2. Bacteriocin loci identified in the genome of strain NT. A schematic representation of gene clusters is shown. Genes are colored by function: putative bacteriocins in red, putative immunity proteins in blue, transporters and associated proteins in dark blue, modification proteins in pink, regulators in light blue, and genes of unknown function in white.

Figure S3

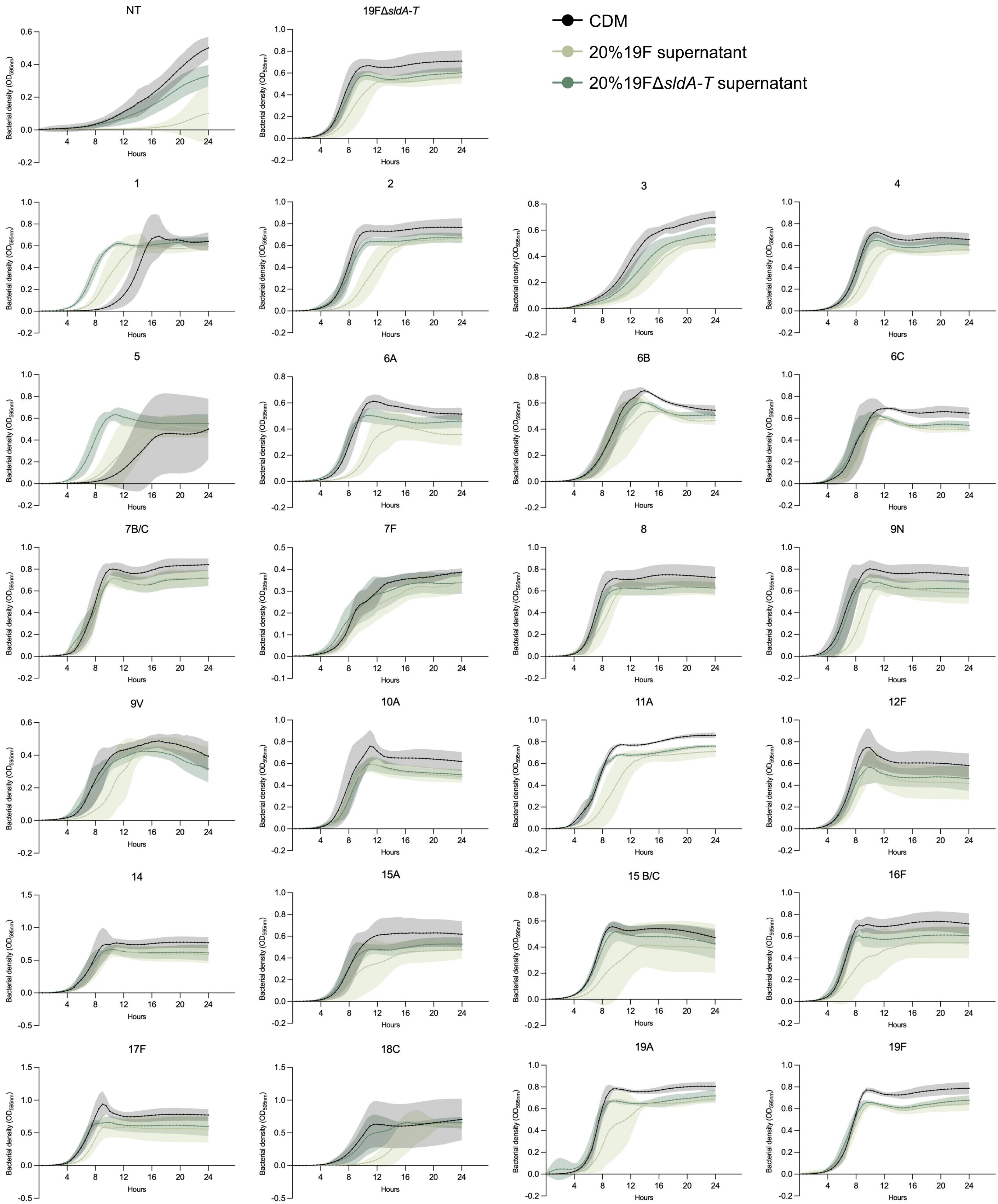

(Continues on the next page)

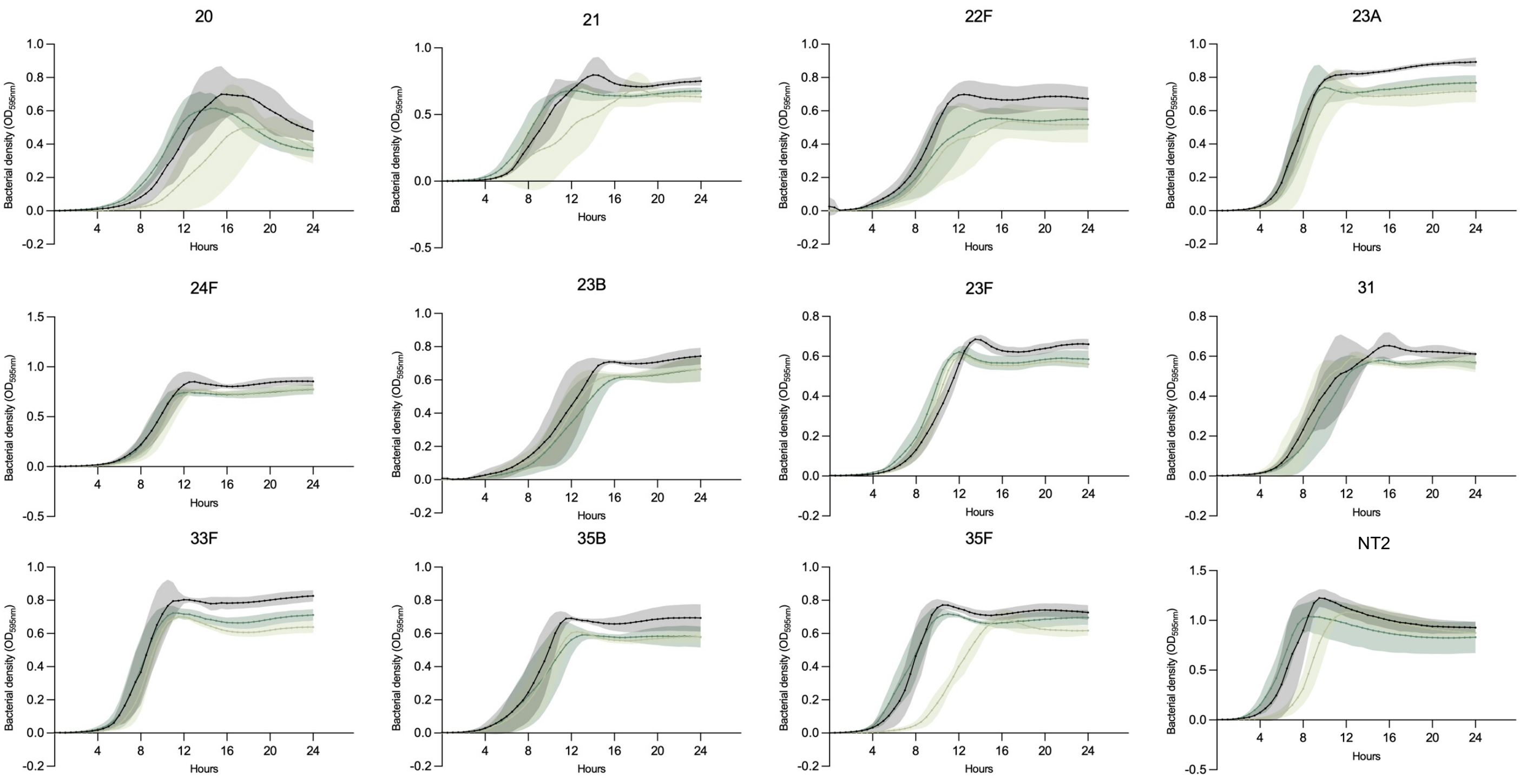

Figure S3: Streptolancidin D - dependent growth inhibition varies across multiple strains. Growth curves of epidemiologically relevant *S. pneumoniae* strains representing serotypes included in pneumococcal conjugate vaccines and non-vaccine types were performed. Strains were grown in CDM in the presence of 20% (v/v) cell-free supernatants of WT (light green) or Δ*sldA-T* (dark green) strains and absence (black). Optical density (OD_{595nm}) was measured at 10 min intervals and plotted. Shaded areas represent standard deviation. Plotted are results of three independent experiments, each with intra-experiment duplicates.

Figure S4

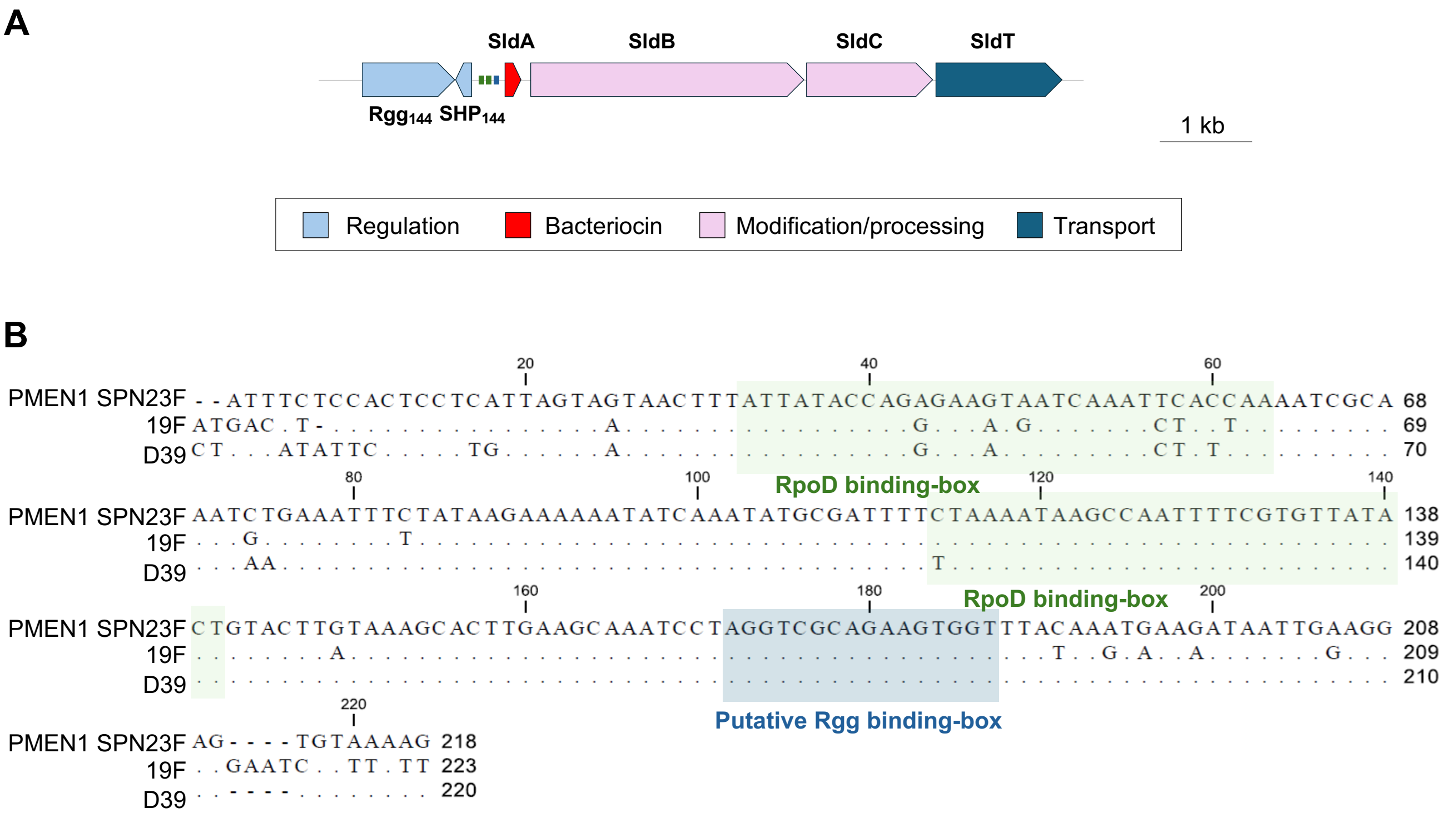

Figure S4. (A) Schematic representation of the *sldA-T* locus and the upstream region containing the Rgg_{144}/SHP_{144} quorum sensing system. Genes are colored by function: bacteriocin in red, transporter in dark blue, modification proteins in pink, and regulators in light blue. Green and blue rectangles indicate RpoD and Rgg binding-boxes. **(B) The intergenic region between SHP_{144} and the first downstream coding sequence in strains 19F, SPN23F and D39 is conserved.** The intergenic region of 19F strain was aligned with that of strains in which functional validation studies of the Rgg/SHP system have been conducted (SPN23F and D39). The putative Rgg binding-box (in blue) predicted by Cuevas et al. is highly conserved in the three strains. Comparison with the genome of strain D39 showed high conservation of RpoD binding-boxes (in green) in strain 19F, as predicted in the Pneumo Browse tool (Slager et al.).

References:

Cuevas RA, Eutsey R, Kadam A, West-Roberts JA, Woolford CA, Mitchell AP, Mason KM, Hiller NL. 2017. A novel streptococcal cell-cell communication peptide promotes pneumococcal virulence and biofilm formation. Mol Microbiol 105:554-571.

Slager J, Aprianto R, Veening JW. 2018. Deep genome annotation of the opportunistic human pathogen *Streptococcus pneumoniae* D39. Nucleic Acids Res 46:9971-9989.

Figure S5

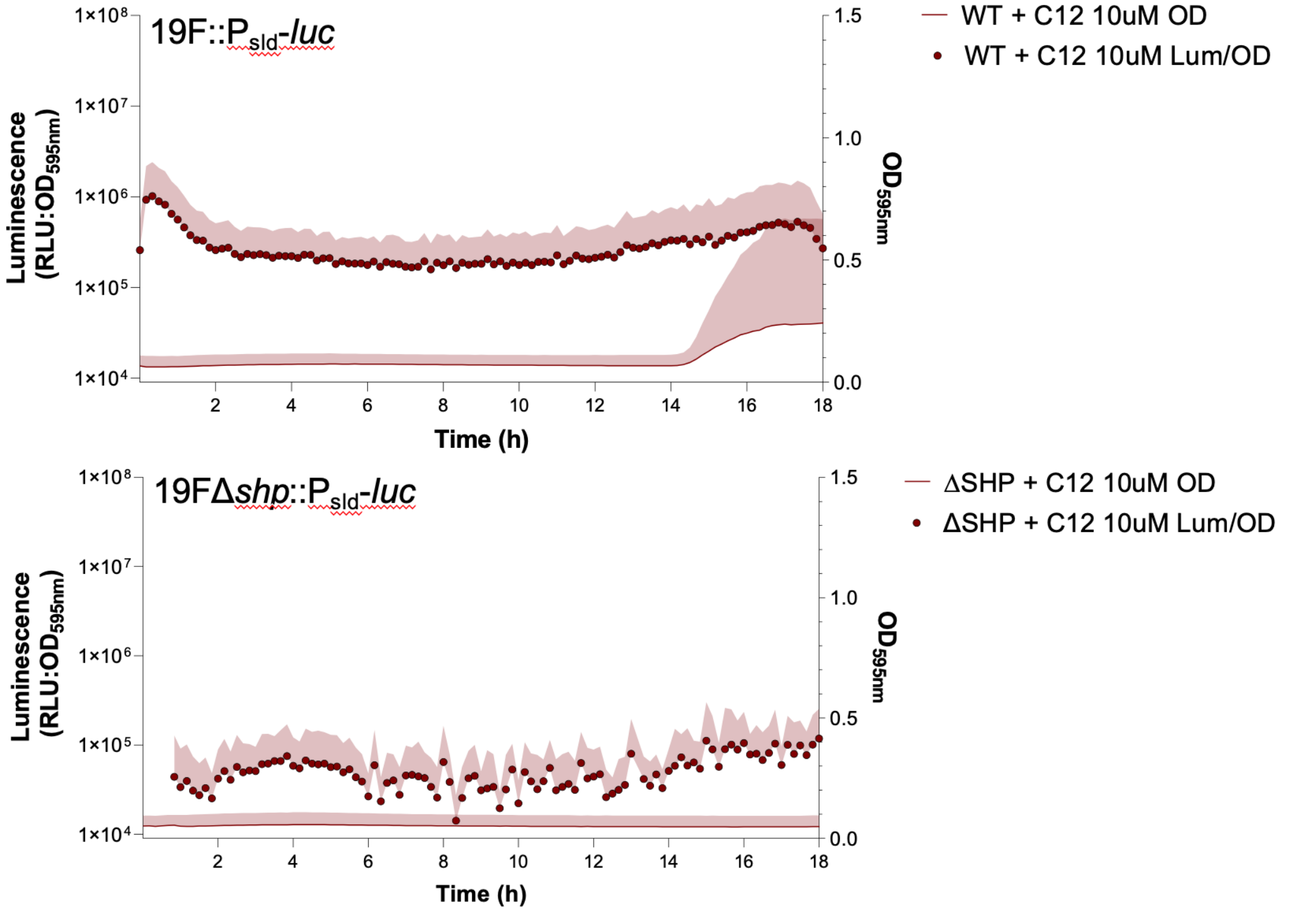

Figure S5. High concentration of SHP impairs growth of 19F strains and does not activate P*sld*. Reporter strains containing the luciferase gene upstream of the ATG of *sldA* gene were constructed in the background of strains 19F' (19F'::P$_{sld}$-*luc*) and 19F'Δ*shp* (19F'Δ*shp*::P$_{sld}$-*luc*). Optical density (OD_{595nm}) and luminescence were mesured at 10 min intervals and plotted. Lines represent growth curves, dots indicate luminescence. Shadded areas represent standard deviation of the mean. Plotted are results of three independent experiments, each with intra-experiment duplicates.
